# Role of the cAMP signaling pathway in the dissemination and development on pepper fruit anthracnose disease caused by *Colletotrichum scovillei*

**DOI:** 10.3389/fcimb.2022.1003195

**Published:** 2022-10-03

**Authors:** Teng Fu, Hyun-Hoo Park, Kyoung Su Kim

**Affiliations:** Division of Bio-Resource Sciences, and Interdisciplinary Program in Smart Agriculture, Kangwon National University, Chuncheon, South Korea

**Keywords:** *Colletotrichum scovillei*, fruit anthracnose, pepper, cAMP signaling, Ca^2+^ signaling

## Abstract

The ascomycete fungus *Colletotrichum scovillei* causes severe anthracnose disease on the fruit of sweet pepper and chili pepper (*Capsicum annuum* L.) worldwide. Understanding the biology of *C. scovillei* would improve the management of fruit anthracnose diseases. The cyclic adenosine monophosphate (cAMP) signaling pathway regulates diverse cellular and physiological processes in several foliar fungal pathogens. We investigated the roles of the cAMP signaling pathway in *C. scovillei* using pharmaceutical and genetic approaches. Exogenous cAMP was found to increase conidiation, appressorium formation, and anthracnose disease development in *C. scovillei*. *CsAc1*, *CsCap1*, and *CsPdeH*, which regulate the intracellular cAMP level, were deleted by homology-dependent gene replacement. Expectedly, the intracellular cAMP level was significantly decreased in *ΔCsac1* and *ΔCscap1* but increased in *ΔCspdeh*. All three deletion mutants exhibited serious defects in multiple fungal developments and pathogenicity, suggesting regulation of the intracellular cAMP level is important for *C. scovillei*. Notably, exogenous cAMP recovered the defect of *ΔCsac1* in appressorium development, but not penetration, which was further recovered by adding CaCl_2_. This result suggests that CsAc1 is associated with both the cAMP and Ca^2+^ signaling pathways in *C. scovillei*. *ΔCscap1* produced morphologically abnormal conidia with reduced tolerance to thermal stress. *ΔCspdeh* was completely defective in conidiation in *C. scovillei*, unlike other foliar pathogens. Taken together, these results demonstrate the importance of cAMP signaling in anthracnose disease caused by *C. scovillei*.

## Introduction

Fungal plant pathogens have evolved diverse and sophisticated signal transduction systems for dispersal and survival in response to the environment and host (Pires-DaSilva and Sommer, 2003; Skerker et al., 2005; De Nadal et al., 2011). Perception of extracellular signals is transferred to second messengers and subsequent downstream regulators (Neves et al., 2002). Cyclic adenosine monophosphate (cAMP), biosynthesized by adenylyl cyclase (Ac) and hydrolyzed by phosphodiesterase (Pde), binds the regulatory subunit of protein kinase A, and triggers its catalytic subunit, thereby modulating downstream signaling and gene transcription (Broach, 1991; Li et al., 2012; Guo et al., 2016; Song et al., 2021).

The cAMP signaling pathway is well known to regulate many aspects of growth and development in filamentous fungi (Adachi and Hamer, 1998; Krüger et al., 1998; Yamauchi et al., 2004; Choi and Xu, 2010; Sassone-Corsi, 2012; Van Zeebroeck et al., 2021). For example, in the rice blast fungus *Magnaporthe oryzae*, deletion of adenylyl cyclase-encoding *MAC1* caused pleiotropic defects, including reduced vegetative growth and conidiation, delayed conidial germination, and complete loss of appressorium formation (Choi and Dean, 1997). The defect in appressorium formation of *MAC1* deletion mutant could be restored by exogenous cAMP (Xu and Hamer, 1996; Kang et al., 1999). The cyclase-associated protein (Cap1), involved in activation of adenylyl cyclase, plays important roles in appressorium development and pathogenicity of *M. oryzae* (Zhou et al., 2012). Deletion of the high-affinity cAMP phosphodiesterase-encoding gene *PdeH* caused increased conidiation, precocious appressorium formation, and defective host colonization (Ramanujam and Naqvi, 2010). In the cucumber anthracnose pathogen *Colletotrichum lagenarium*, CAC1 and Cpk1 are essential for virulence, conidial germination, and lipid degradation during appressorium development (Yamauchi et al., 2004). In the maize head blight pathogen *Fusarium graminearum*, Fgac1 and FgCap1 regulate development, virulence, and mycotoxin production (Bormann et al., 2014; Yin et al., 2018). Therefore, cAMP–PKA signaling is indispensable for the differentiation and pathogenicity of plant-pathogenic fungi.

The *Colletotrichum acutatum* species complex causes severe anthracnose diseases in hundreds of plant species of over 90 genera worldwide (Baroncelli et al., 2017). *C. scovillei*, a member of the *C. acutatum* species complex, is the major causal agent of fruit anthracnose in pepper (*Capsicum annuum* L.) in tropical and subtropical regions (Fu et al., 2021). The polycyclic pathogen *C. scovillei* produces massive conidia that propagate fruit anthracnose throughout the pepper growth season (Fu et al., 2022). Upon recognition of host physical and chemical signals, *C. scovillei* conidium germinates and develops an appressorium at the tip of the germ tube (Gao et al., 2018). The appressorium penetrates the host epidermis *via* a penetration peg and differentiates into invasive hyphae to infect host tissues (Fu et al., 2021). During appressorium-mediated penetration, a unique dendroid structure develops in the cuticle layer of pepper fruit, which does not occur in foliar infections by other fungal pathogens (Liao et al., 2012; Saxena et al., 2016). The cAMP signaling pathway has been characterized in several foliar pathogens (Ebbole, 2007; Dean et al., 2012; Kazan et al., 2012; Aramayo and Selker, 2013), but not in the pepper fruit anthracnose pathogen *C. scovillei*.

To assess the role of the cAMP signaling pathway in fungal development and the pathogenicity of *C. scovillei*, we evaluated the effect of exogenous cAMP on mycelial growth, conidiation, appressorium formation, and infection of the wild-type. Exogenous cAMP enhanced conidiation, appressorium formation, and plant infection in *C. scovillei*. To investigate the roles of components of the cAMP signaling pathway, we deleted in *C. scovillei CsAc1*, *CsCap1*, and *CsPdeH*, encoding an adenylyl cyclase, adenylyl cyclase-associated protein, and high-affinity cAMP phosphodiesterase, respectively. *CsAc1*, *CsCap1*, and *CsPdeH* have overlapping and distinct functions in development and anthracnose disease caused by *C. scovillei*. Deletion of *CsAc1* abolished appressorium formation, penetration, and invasive hyphal growth. Conidia of the *CsCap1* deletion mutant were morphologically abnormal, hypersensitive to thermal stress, and reduced pathogenicity. The *CsPdeH* deletion mutant failed to produce conidia, but infected pepper fruit with reduced severity *via* mycelia. Our results indicate important roles for cAMP signaling in *C. scovillei*, providing insight into the molecular mechanisms of fruit anthracnose diseases caused by *Colletotrichum* fungi.

## Materials and methods

### Fungal strains, culture conditions, and bioinformatic tools

The wild-type *C. scovillei* (strain KC05) and its transformants were routinely grown on V8 juice agar medium (V8A), potato dextrose agar medium (PDA), and oatmeal agar medium (OMA) (Fu et al., 2022). Mycelia used for extraction of DAN and RNA were cultured in liquid complete medium (CM) and on TB3 agar medium (Lee et al., 2021). Sequences of *CsAc1, CsCap1, CsPdeH* and their homologs were obtained from online databases of Comparative Fungal Genomics (http://cfgp.riceblast.snu.ac.kr) and National Center for Biotechnology Information (http://www.ncbi.nlm.nih.gov). Sequences were aligned using MEGA X and illustrated using BioEdit 7.2.

### Targeted gene deletion and complementation

A modified double-joint PCR was used to fuse DNA segments for generation of target deletion mutants as previously described (Yu et al., 2004; Shin et al., 2019). DNA fragments (about 1.5 kb) from both upstream and downstream of target genes were amplified with the primers 5F/5R and 3F/3R of each corresponding gene, respectively (**Supplementary Table S1**). The *hygromycin B phosphotransferase* (*HPH*) was fused with upstream and downstream segments of each gene by rounds of fusion PCR with corresponding primers 5F/3R (**Supplementary Table S1)**. The fused constructs were amplified with corresponding primers NF/NR (**Supplementary Table S1**) and next transformed into wild-type protoplasts. Obtained transformants were grown on TB3 agar medium and screened with screening PCR with corresponding primers SF/SR (**Supplementary Table S1**). The purified progenies of candidate transformants were selected *via* Southern blotting. The complemented strains were generated by co-introducing the geneticin-resistant cassette and segments amplified with primers NF/NR (**Supplementary Table S1**) of each corresponding gene from wild-type genome into protoplasts of corresponding deletion mutants. Complemented strains were selected with screening PCR.

### Nucleic acid manipulation, RT-PCR, and Southern blotting

Fungal genomic DNA was isolated *via* a quick extraction method used for screening PCR and a standard method used for Southern blotting (Chi et al., 2009; Han et al., 2018). For southern blotting, genomic DNA was digested with specific restriction enzyme and then probed with a DNA segment (500 bp), which was amplified with primers PF/PR (**Supplementary Table S1**) and labeled with Biotin-High Prime (Roche, IN). To perform RT-PCR, total RNA was extracted from frozen fungal mycelia using an Easy-Spin Total RNA extraction kit (iNtRON Biotechnology, South Korea). The complementary DNA (cDNA) was reversely transcribed from 5 µg of total RNA using SuperScript III first-strand synthesis kit (Invitrogen, Invitrogen, CA). The *β-tubulin* (CAP_007327) was used as a reference gene in RT-PCR and qRT-PCR. The qRT-PCR was performed using the StepOne real-time PCR system (Applied Biosystems, Foster city, CA) with two replicates in three independent experiments. The relative gene expression was calculated as 2*^−ΔΔCT^* (Livak and Schmittgen, 2001; Fu et al., 2022).

### Measurement of intracellular cAMP concentration

All strains were cultured in liquid CM broth and shaken at 150 rpm and 25°C for 3 days. The fresh mycelia were firstly filtered through two layers of miracloth (Calbiochem, CA) and then powdered with liquid nitrogen. The intracellular concentrations of cAMP were measured using a cAMP complete ELISA kit (Enzo Life Sciences, Farmingdale, NY) according to manufacturer’s instructions. For each sample, 0.1 g of fine powder were homogenized in 1 mL of 0.1 M HCl. Intracellular concentrations of cAMP were measured in three independent experiments with three replicates.

### Fungal development assays

Mycelial growth was evaluated by measuring colony diameters on CMA and MMA at 5 days. Hyphae grown on MMA were stained with calcofluor white to visualize septa under a fluorescent microscope. Conidiation was evaluated by counting the number of conidia in 7-day-old V8 agar with 5 mL distilled H_2_O. To distinguish conidiophores, lactophenol aniline blue solution was used to stained mycelia (Fu et al., 2021). Appressorium formation were performed by dropping conidial suspensions (5 × 10^4^/mL), harvested from 7-day-old OMA, on the hydrophobic surface of coverslips and the hydrophilic surface of slid glasses (Waldemar Knittel Glasbearbeitungs, Germany), and the surface of pepper fruits. The percentage of appressorium formation were calculated by counting the number of appressorium-forming conidia in a total of 100 conidia. All data were collected from three independent experiments with triplicates per experiment.

### Plant infections assays

For plant infection assays, conidia suspensions (50 × 10^4^/mL), harvested from 7-day-old OMA, were inoculated onto intact and wounded pepper fruits, and incubated in humid plastic boxes at 25°C. Lesion sizes of anthracnose disease on pepper fruits were measured by using Image J. To observe infectious hyphae, thin sections were sliced using a razor blade from infected pepper fruits and then immersed in mixture of acetic acid, chloroform, and methanol (Fu et al., 2022). The samples were subsequently rehydrated in ethanol solutions of decreasing concentrations, finally stained in modified trypan blue solution (Fu et al., 2021).

## Results

### Effect of cAMP on the development and anthracnose disease of *C. scovillei*

**Figure 1:**
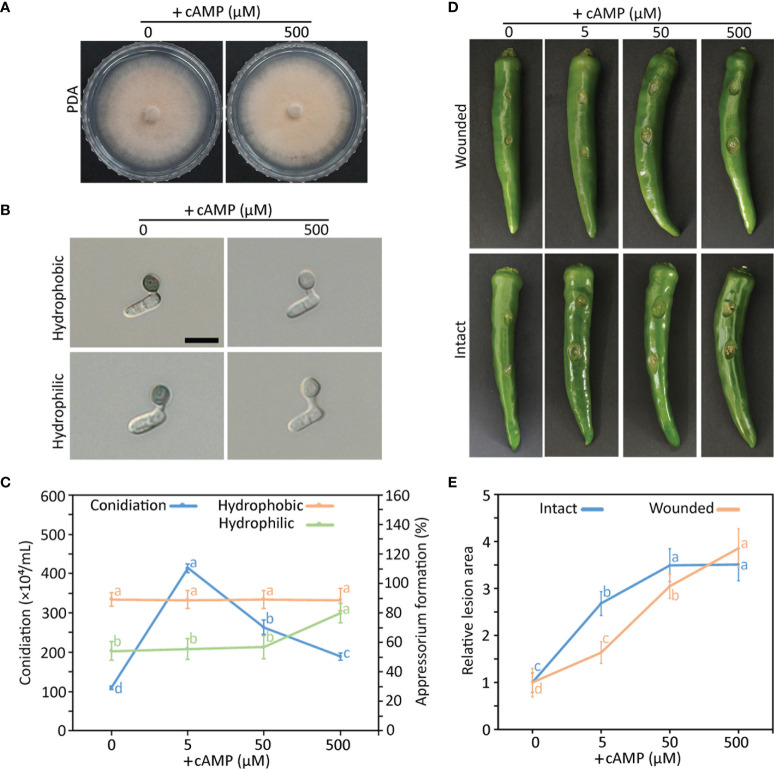
Effect of exogenous cAMP on mycelial growth **(A)**, appressorium formation **(B, C)**, conidiation **(C)**, pathogenicity **(D, E)** of wild-type *C scovillei*. **(A)** Mycelial growth. Mycelial agar plugs (5-mm diameter) from 4-day-old MMA were inoculated onto potato dextrose agar (PDA) containing 0 and 500 µM cAMP and cultured for 6 days. **(B)** Appressorium formation. Conidial suspensions (5 × 10^4^/mL) were dropped onto the hydrophobic surface of coverslips and slide glasses and incubated in a humid plastic box for 16 hours. Scale bar, 10 μm. **(C)** Quantitative measurements of conidiation and appressorium formation. Mycelial agar plugs (5 mm diameter) from 4-day-old MMA were inoculated onto PDA containing 0, 5, 50, and 500 µM cAMP and cultured under a cycle of 6 days of light and 2 days of darkness. Conidia were harvested in 5 mL of distilled H_2_O. Appressorium formation was measured by counting appressorium-forming conidia among a total of 100 conidia. **(D, E)** Pathogenicity assays. Intact and wounded pepper fruits were inoculated with conidial suspensions (50 × 10^4^/mL) and incubated in a humid plastic box. After 24 hours, 0, 5, 50, and 500 µM cAMP was added to conidial drops. **(D)** Photographs of wounded and intact pepper fruits after 6 and 8 days, respectively. **(E)** Anthracnose lesion size measured using ImageJ. Error bars show standard deviations and different letters in the same group indicate a significant difference estimated using Duncan’s test (P < 0.05).

To investigate the role of the cAMP signaling pathway in the differentiation and pathogenicity of *C. scovillei*, we evaluated mycelial growth, conidiation, appressorium formation, and plant infection with exogenous treatments of elevated concentrations of cAMP. Exogenous cAMP did not influence mycelial growth (**Figure 1A**). However, conidiation, appressorium formation, and plant infection were enhanced by exogenous cAMP (**Figures 1B–E**). Interestingly, appressorium melanization was suppressed by exogenous cAMP on artificial surfaces (**Figure 1B** and **Supplementary Table S2**). These results suggest that the cAMP signaling pathway is important for the differentiation and pathogenicity of *C. scovillei*.

### Phylogenetic analysis and sequence alignments of *CsAc1, CsCap1*, and *CsPdeH*

We next functionally characterized *CsAc1* (*CAP_008564*), *CsCap1* (CAP_009109), and *CsPdeH* (CAP_005557), which encode Ac1, Cap1, and PdeH, respectively, known for modulating intracellular cAMP concentration (Choi and Dean, 1997; Ramanujam and Naqvi, 2010; Zhou et al., 2012; Yin et al., 2018; Guo et al., 2019). *CsAc1, CsCap1*, and *CsPdeH* were isolated from *C. scovillei* by individually blasting MAC1 (XP_003709992.1), Cap1 (XP_003714719.1), and PdeH (XP_003710562.1). Phylogenetic analysis showed that *CsAc1, CsCap1*, and *CsPdeH* were closely related to orthologs in filamentous fungi, and distantly related to orthologs in yeasts (**Supplementary Figure S1**). Domain predictions showed that all orthologs of Ac1, Cap1, and PdeH contained four domains (adenylyl cyclase G-alpha binding, Ras-associating protein, PPM-type phosphate domain, and adenylyl cyclase class-3/4 guanylyl cyclase), two domains (adenylyl cyclase-associated CAP, N/C terminal), and one domain (3’5’-cyclic nucleotide phosphodiesterase, cyclitic domain), respectively (**Supplementary Figure S1**). These results suggest that Ac1, Cap1, and PdeH are highly conserved in filamentous fungi.

### Modulation of the intracellular cAMP level by *CsAc1*, *CsCap1*, and *CsPdeH*

**Figure 2:**
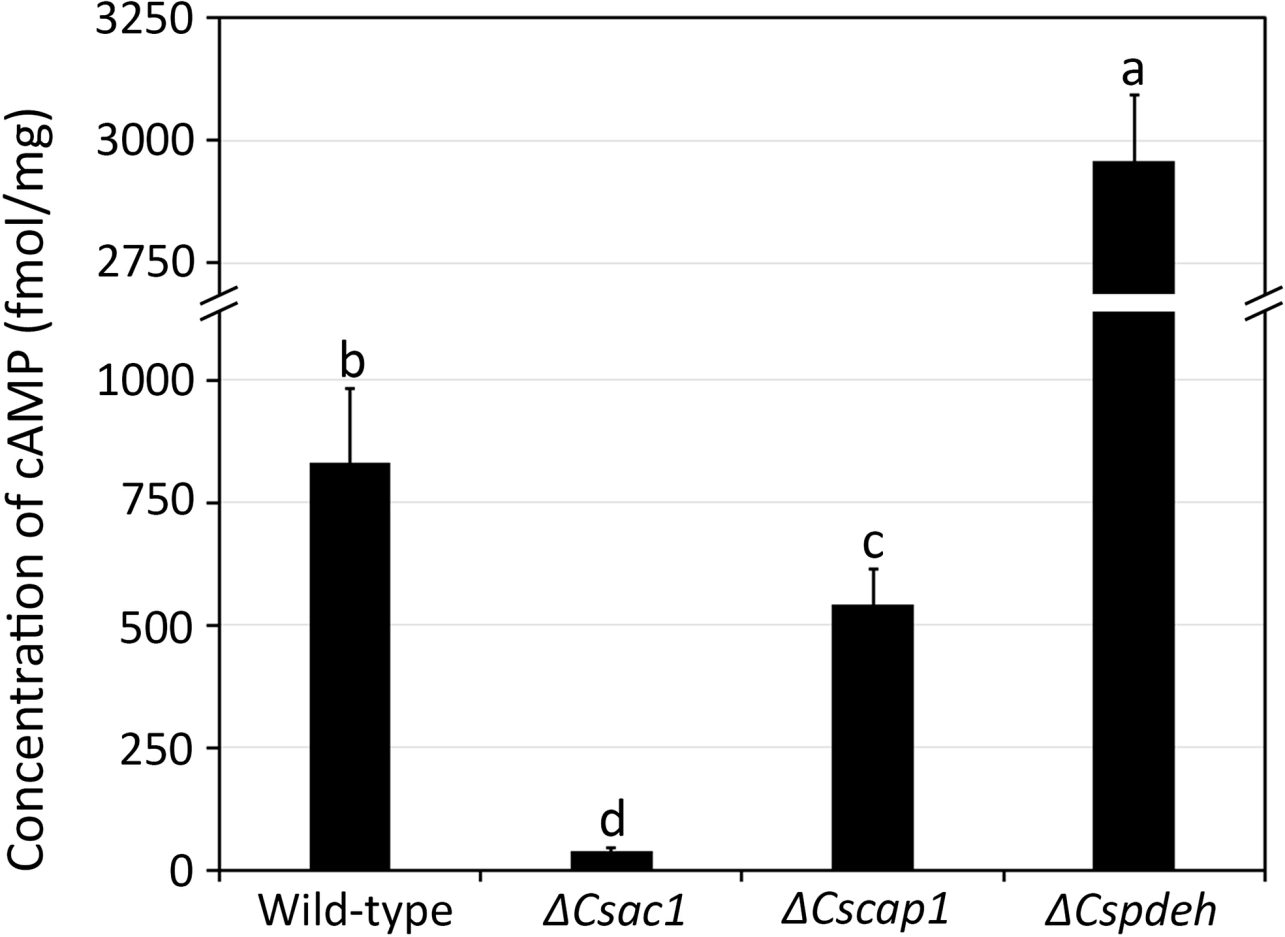
Quantitative measurement of intracellular cAMP concentration. Wild-type, *ΔCsac1*, *ΔCscap1*, and *ΔCspdeh* were inoculated into 50 mL of CM broth and rotated at 150 rpm and 25°C for 3 days. The intracellular cAMP level was measured using 100 mg of mycelial powder. Error bars show standard deviations and different letters in the same group indicate significant differences estimated using Duncan’s test (P < 0.05).

To investigate roles the of *CsAc1*, *CsCap1*, and *CsPdeH* in *C. scovillei*, we deleted three targeted genes individually *via* homologous replacement (**Supplementary Figure S2**). The correct deletion mutants were confirmed by Southern blotting (**Supplementary Figure S2**). The expression of each target gene was detected in the wild-type and the corresponding complemented strain, but not in the corresponding deletion mutant, by RT-PCR (**Supplementary Figure S2**). To determine whether *CsAc1*, *CsCap1*, and *CsPdeH* are involved in modulating the intracellular cAMP level, we assayed the cAMP concentration in mycelia cultured in CM broth (**Figure 2**). The intracellular cAMP concentration was significantly lower in *ΔCsac1* (38.2 ± 8.7 fmol/mg) and *ΔCscap1* (540.6 ± 75.6 fmol/mg), but markedly higher in *ΔCspdeh* (2957.2 ± 133.9 fmol/mg), compared to the wild-type (831.8 ± 150.8 fmol/mg). This result suggests that *CsAc1, CsCap1*, and *CsPdeH* modulate the intracellular cAMP concentration of *C. scovillei*.

### Roles of *CsAc1*, *CsCap1*, and *CsPdeH* in mycelial growth

**Figure 3:**
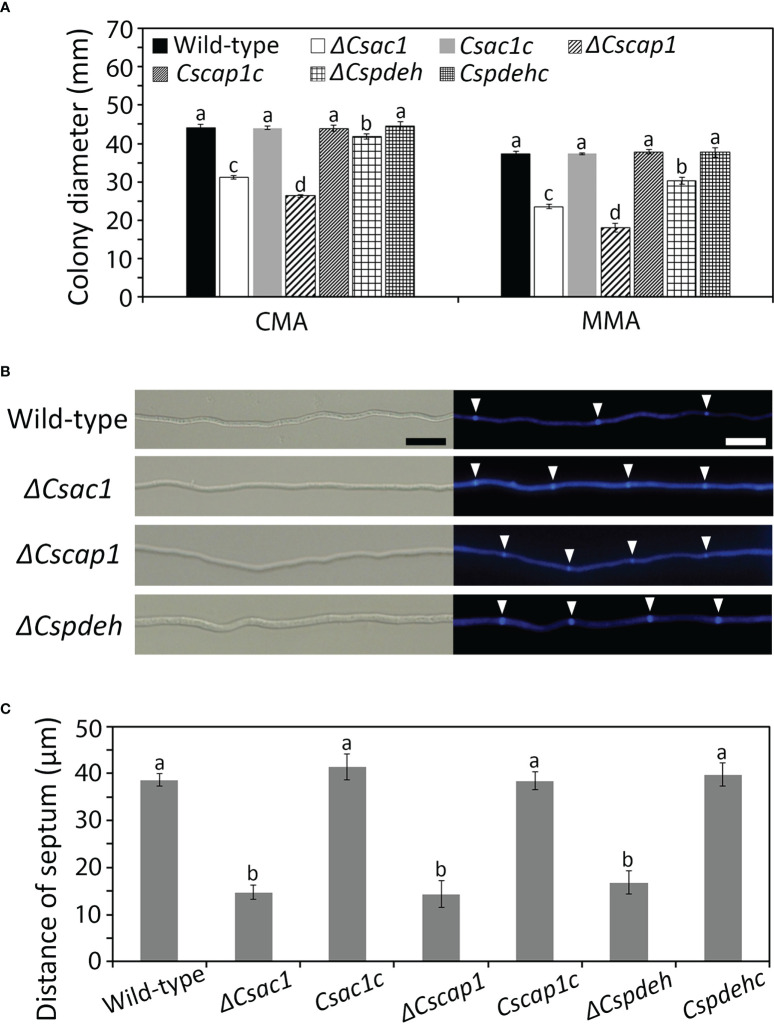
Roles of *CsAc1*, *CsCap1*, and *CsPdeH* in vegetative growth. **(A)** Quantitative measurement of mycelial growth. Mycelial agar plugs (5 mm diameter) from 4-day-old MMA were inoculated onto CMA and MMA and incubated at 25°C without light. Colony diameters were measured after 5 days. **(B, C)** Increased septation during vegetative growth of *ΔCsac1*, *ΔCscap1*, *ΔCspdeh*. **(B)** Septa in mycelia at 24 hours post-inoculation on CMA with calcofluor white staining. Scale bar, 20 μm. **(C)** Septum distance measured using ZEN imaging software. For each strain, 50 hyphal compartments were measured. Error bars show standard deviations and different letters in the same group indicate significant differences estimated using Duncan’s test (P < 0.05).

To determine whether *CsAc1*, *CsCap1*, and *CsPdeH* are involved in mycelial growth, colony diameters on CMA and MMA were measured. The colony diameter was significantly smaller in *ΔCsac1* (CMA: 31.2 ± 0.4 mm; MMA: 23.4 ± 0.5 mm), *ΔCscap1* (CMA: 26.3 ± 0.4 mm; MMA: 18.2 ± 1.1 mm), and *ΔCspdeh* (CMA: 41.2 ± 0.8 mm; MMA: 30.3 ± 0.9 mm), compared to the wild-type (CMA: 44.2 ± 0.8 mm; MMA: 37.3 ± 0.8 mm) (**Figure 3A** and **Supplementary Figure S3**). To investigate the defect in mycelial growth of deletion mutants, we measured the distances between hyphal septa. The hyphal compartment length was significantly shorter in *ΔCsac1* (14.7 ± 1.5 µm)*, ΔCscap1* (14.3 ± 2.8 µm), and *ΔCspdeh* (16.8 ± 2.4 µm), compared to the wild-type (38.6 ± 1.4 µm) (**Figures 3B, C**). The defects in colony growth and hyphal septation of deletion mutants were restored in the corresponding complemented strains (**Figures 3A–C**), suggesting that *CsAc1*, *CsCap1*, and *CsPdeH* are involved in hyphal growth.

### Roles of *CsAc1*, *CsCap1*, and *CsPdeH* in conidium production, morphology, and viability

**Figure 4:**
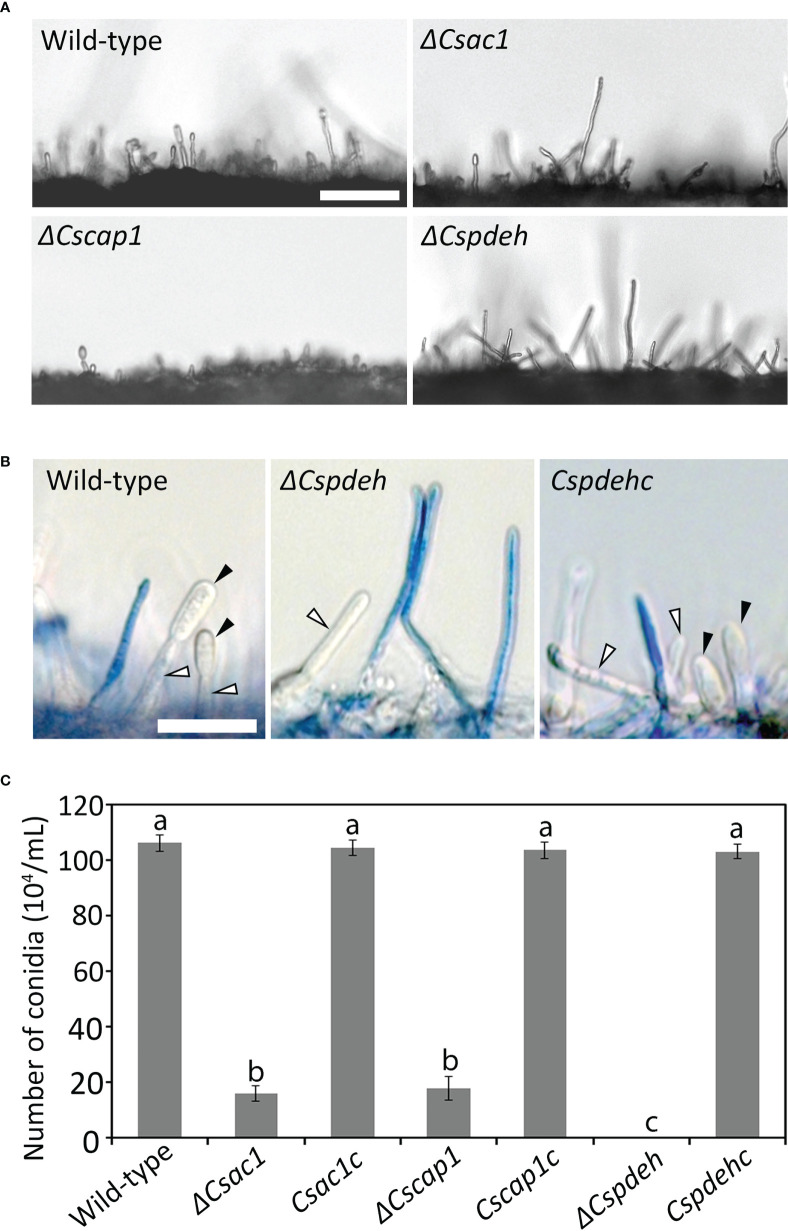
Roles of *CsAc1*, *CsCap1*, and *CsPdeH* in conidiation. **(A)** Microscopic visualization of conidiation. The indicated strains were inoculated onto oatmeal agar (OMA) and incubated for 3 days without light. Mycelial agar plugs from 3-day-old OMA were placed in a humid plastic box and incubated with light for 5 hours. Scale bar, 50 μm. **(B)** Microscopic visualization of conidiophores under inductive conditions. Aerial hyphae (blue) other than conidiophores were stained using lactophenol aniline blue solution. Black and white triangles indicate conidia and conidiophores, respectively. Scale bar, 20 μm. **(C)** Quantitation of conidiation. The indicated strains were inoculated into PDA and incubated under a cycle of 5 days of darkness and 2 days light. Error bars show standard deviations and different letters in the same group indicate significant differences estimated using Duncan’s test (P < 0.05).

**Figure 5:**
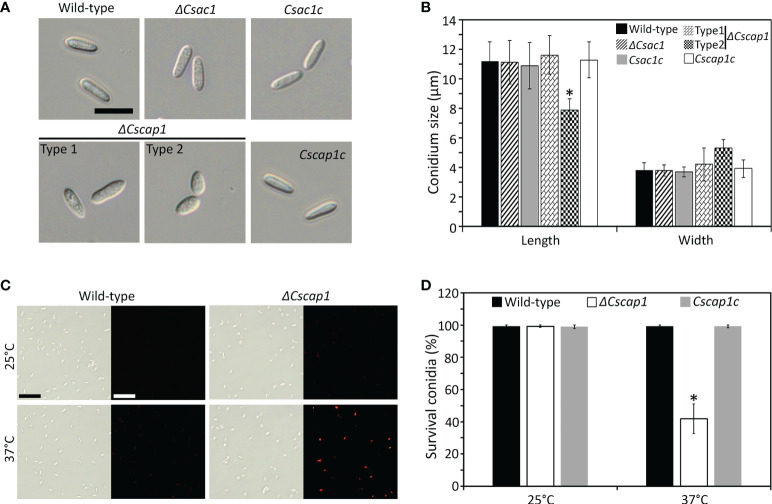
Roles of *CsAc1*, *CsCap1*, and *CsPdeH* in conidium morphology and conidium viability. **(A, B)** Visualization and measurement of conidium morphology. **(A)** Conidia were collected from 7-day-old OMA cultures using distilled H_2_O. Scale bar, 10 μm. **(B)** Length and width of 100 conidia. *ΔCscap1* produced two types (types 1 and 2) of conidia. **(C, D)** Conidium viability under heat shock. **(C)** Conidia stained with phloxine **(B)** Conidia of the indicated strains were incubated at 25°C or 37°C for 16 hours and stained with phloxine **(B)** Survival is indicated by red fluorescence. Scale bar, 50 μm. **(D)** Quantitation of conidium survival (n = 100 conidia). Error bars show standard deviations and asterisk (*) indicates a significant difference estimated using Duncan’s test (P < 0.05).

To assess the roles of *CsAc1*, *CsCap1*, and *CsPdeH* in conidiogenesis, we evaluated conidiation. Conidiation was significantly reduced in *ΔCsac1* [(16.7 ± 2.7) × 10^4^ conidia/mL] and *ΔCscap1* [(17.9 ± 4.3) × 10^4^ conidia/mL] compared to the wild-type [(66.4 ± 3.1) × 10^4^ conidia/mL] (**Figures 4A–C**). Notably, *ΔCspdeh* abolished conidiation (**Figures 4A, C**). However, *ΔCspdeh* developed conidiophores according to lactophenol aniline blue staining assay (**Figure 4B**). The defects of conidiation in the deletion mutants were recovered in the corresponding complemented strains (**Figures 4A–C**). This result suggests that *CsAc1* and *CsCap1* are quantitatively related to conidiation and that *CsPdeH* is essential for conidia differentiation from conidiophores in *C. scovillei*. Interestingly, conidia produced by *ΔCscap1* were morphologically abnormal (**Figures 5A, B**). Because the morphologically abnormal conidia produced by deletion mutants of *C. scovillei* have altered survival under heat shock stress (Fu et al., 2021; Fu et al., 2022), we examined conidium survival of *ΔCscap1.* Conidium survival of *ΔCscap1* was normal at 25°C, but significantly reduced at 37°C, compared to the wild-type and *Cscap1c* (**Figures 5C, D**). Taken together, these results suggest that *CsAc1*, *CsCap1*, and *CsPdeH* are important for conidiation and *CsCap1* is related to conidium morphology and survival in *C. scovillei*.

### Roles of *CsAc1, CsCap1*, and *CsPdeH* in appressorium development

**Figure 6:**
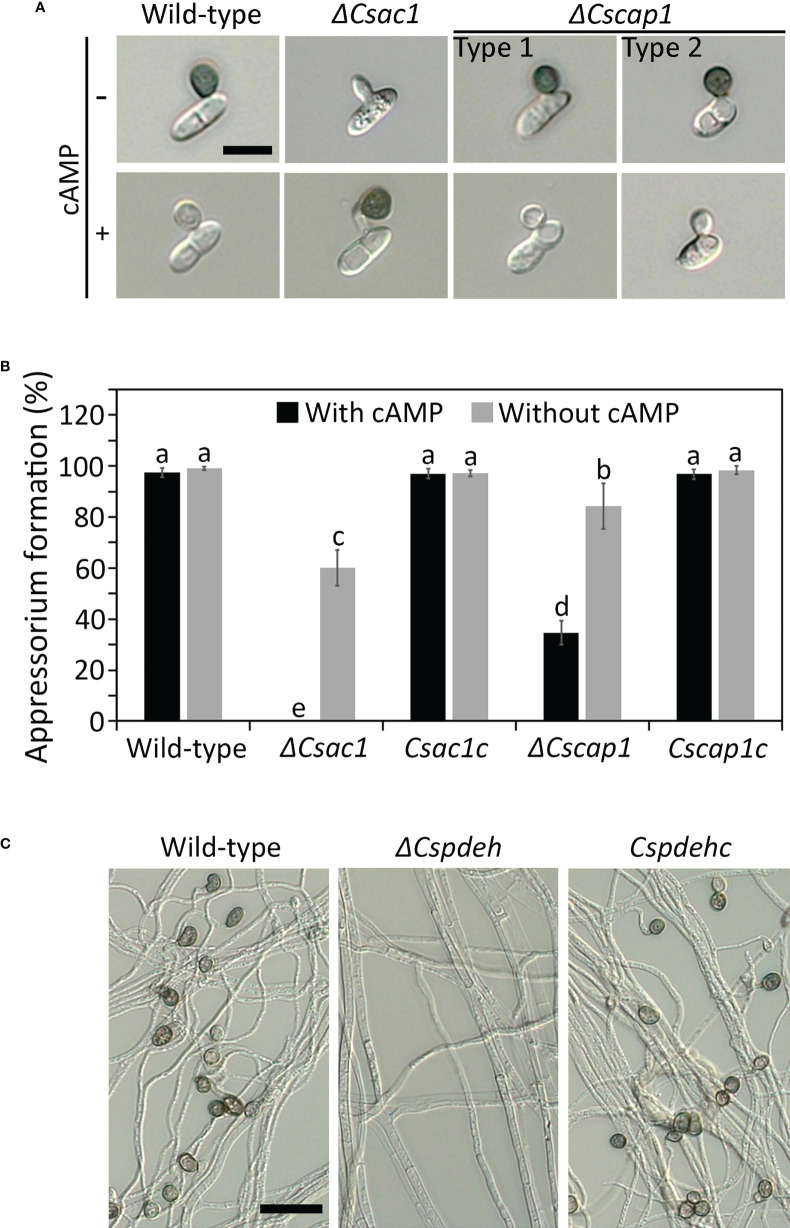
Roles of *CsAc1*, *CsCap1*, and *CsPdeH* in appressorium formation. **(A, B)** Appressorium developed by conidia of *ΔCsac1* and *ΔCscap1*. **(A)** Conidial suspensions (5 × 10^4^/mL) were dropped onto the hydrophobic surface of coverslips. After 12 hours, exogenous cAMP (2.5 mM) was added to conidial drops and incubated for 12 hours. Scale bar, 10 μm. **(B)** Quantitation of appressorium formation by conidia (n = 100 conidia). **(C)** Appressorium-like structure formation of *ΔCspdeh*. Mycelial agar plugs from 3-day-old OMA cultures were placed on the hydrophobic surface of coverslips and incubated in a humid box for 4 days. Scale bar, 20 μm. Error bars show standard deviations and different letters in the same group indicate significant differences estimated using Duncan’s test (P < 0.05).

To investigate the roles of *CsAc1* and *CsCap1* in appressorium development, we monitored appressorium formation on the hydrophobic surface of coverslips. After 24 hours, the appressorium formation rate was significantly lower in *ΔCscap1* (34.7 ± 4.7%) than the wild-type (97.5 ± 0.8%) (**Figures 6A, B**). *ΔCsac1* showed abolished appressorium development (**Figures 6A, B**). The defect in appressorium formation was recovered in the complemented strains *Csac1c* and *Cscap1c* (**Figures 6A, B**). This result suggests that that *CsAc1* is essential for, and *CsCap1* is quantitatively related to, appressorium formation of *C. scovillei*. We further attempted to restore appressorium formation using exogenous cAMP. Exogenous addition of 2.5 mM cAMP significantly enhanced appressorium formation in *ΔCsac1* (60.2 ± 7.1%) and *ΔCscap1* (84.3 ± 9.0%) (**Figures 6A, B**). In contrast to the wild-type and *Csac1c*, appressoria of *ΔCsac1* were melanized by exogenous cAMP (**Figure 6A**), suggesting that cAMP partially restored appressorium formation of *ΔCsac1*. Because *ΔCspdeh* is unable to produce conidia, we examined appressorium-like structure development from hyphal tips. Hyphal tips of the wild-type and *Cspdehc* developed many appressorium-like structures (**Figure 6C**). However, *ΔCspdeh* failed to differentiate any appressorium-like structure from the hyphal tip (**Figure 6C**), suggesting that *CsPdeH* is required for appressorium-like structure formation in *C. scovillei*.

### Roles of *CsAc1, CsCap1*, and *CsPdeH* in anthracnose development

**Figure 7:**
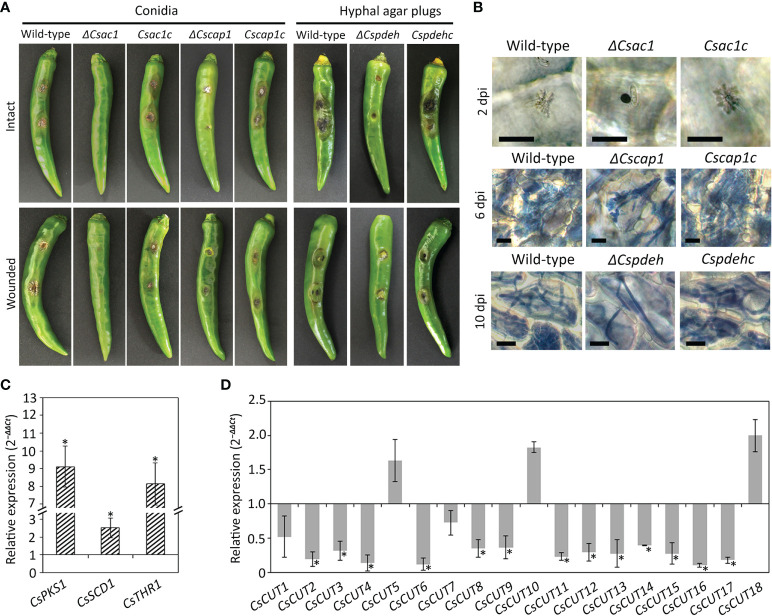
Roles of *CsAc1*, *CsCap1*, and *CsPdeH* in plant infection. **(A)** Photographs of anthracnose disease formation. Conidial suspensions (50 × 10^4^/mL) were dropped onto intact and wounded pepper fruits and incubated in a humid plastic box at 25°C for 9 and 6 days, respectively. Hyphal agar plugs from 3-day-old OMA cultures were inoculated onto intact and wounded pepper fruits and incubated in a humid plastic box at 25°C for 11 and 8 days, respectively. **(B)** Microscopic visualization of infection. Conidial suspensions (5 × 10^4^/mL) were dropped onto intact pepper fruits. Pepper fruits infected with the indicated strains showed dendroid structures in the cuticle layers. Invasive hyphae were stained blue using modified trypan blue solution. Scale bar, 20 μm. **(C, D)** Expression levels of melanin synthesis and cutinase genes. The *C. scovillei* β‐tubulin gene was used as a reference gene. The targeted genes in the wild-type were expressed as a relative of 1. Asterisk (*) indicates a significant difference. **(C)** Expression of melanin synthesis genes in *ΔCsac1* compared to the wild-type. Melanin synthesis genes includes *CsPKS1* (*CAP_001057*), *CsSCD1* (*CAP_008724*), and *CsTHR1* (*CAP_008847*). **(D)** Expression of cutinase genes in pepper fruit tissues infected with *ΔCsac1* compared to the wild-type. Information of cutinase genes is shown in **Supplementary Table S3**.

To assess the roles of *CsAc1*, *CsCap1*, and *CsPdeH* in anthracnose development, we inoculated intact and wounded pepper fruits with conidial suspensions of *ΔCsac1* and *ΔCscap1*, and mycelial agar plugs of *ΔCspdeh*. The wild-type and complemented strains formed typical anthracnose lesions on intact and wounded pepper fruits (**Figure 7A**). However, *ΔCsac1* failed to cause anthracnose disease on intact and wounded pepper fruits (**Figure 7A**). *ΔCscap1* and *ΔCspdeh* reduced abilities to cause anthracnose disease on wounded pepper fruits (**Figure 7A**). These results suggest that *CsAc1* is essential for, and *CsCap1* and *CsPdeH* are associated with, plant infection by *C. scovillei*. By microscopy, *ΔCsac1* developed melanized appressoria, which failed to grow invasive hyphae, whereas the wild-type penetrated and induced dendroid structures in the cuticle layers of pepper fruits (**Figure 7B**). This result suggests that *CsAc1* is essential for appressorium-mediated penetration by *C. scovillei*. We further evaluated the expression levels of melanin synthesis genes and plant cell wall-degrading enzymes, such as cutinases by qRT-PCR. Melanin synthesis genes, including *CsPKS1*, *CsSCD1*, and *CsTHR1*, were highly expressed and 13 cutinase genes were greatly reduced in *ΔCsac1* compared to the wild-type (**Figures 7C, D** and **Supplementary Table S3**), suggesting that CsAc1 positively regulates the expression of cutinases and negatively regulates the expression of melanin synthesis genes. *ΔCscap1* and *ΔCspdeh* produced fewer invasive hyphae than the wild-type (**Figure 7B**), suggesting that *CsCap1* and *CsPdeH* are associated with invasive hyphal growth in *C. scovillei*. Collectively, these results suggest that *CsAc1* is essential for both appressorium-mediated penetration and invasive hyphal growth, and that *CsCap1* and *CsPdeH* are associated with invasive hyphal growth of *C. scovillei*.

### Restoration of the penetration defect in *ΔCsac1* by exogenous cAMP and CaCl_2_

**Figure 8:**
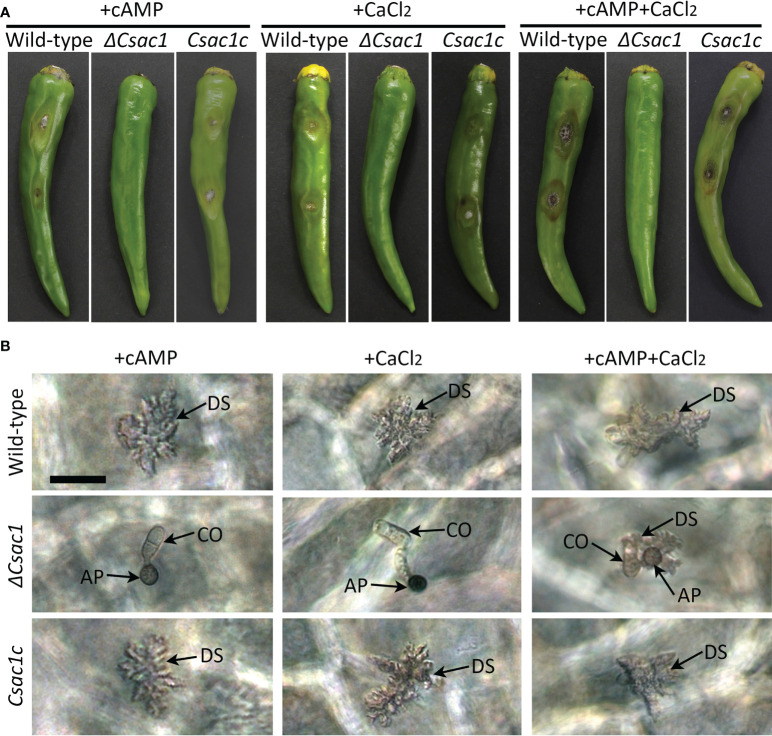
Recovery of appressorium-mediated penetration of *ΔCsac1* by exogenous cAMP and CaCl_2_. **(A)** Pathogenicity of *ΔCsac1*. Intact and wounded pepper fruits were inoculated with conidial suspensions (50 × 10^4^/mL) and incubated in a humid plastic box. After 24 hours, 2.5 mM cAMP, 15 µM CaCl_2_, and 2.5 mM cAMP and 15 µM CaCl_2_ were added to conidial drops. Photographs were taken after 9 days. **(B)** Microscopic observation of penetration by *ΔCsac1*. Conidial suspensions (5 × 10^4^/mL) were dropped onto pepper fruits and incubated in a humid plastic box. After 24 hours, 2.5 mM cAMP, 15 µM CaCl_2_, and 2.5 mM cAMP and 15 µM CaCl_2_ were added. Photographs were taken after 3 days. Scale bar, 20 μm.

Because exogenous cAMP partially restored appressorium development by *ΔCsac1* on a hydrophobic surface, we investigated the effect of exogenous cAMP on plant infection by *ΔCsac1*. Unexpectedly, *ΔCsac1* failed to cause anthracnose, whereas the wild-type and *Csac1c* caused typical anthracnose lesions on intact pepper fruits with exogenous cAMP (**Figure 8A**). Microscopic observation showed that appressoria of *ΔCsac1* induced by exogenous cAMP failed to penetrate host tissues (**Figure 8B**). Because the Ca^2+^ signaling pathway is important for appressorium-mediated penetration in *M. oryzae* (Choi et al., 2011), we next evaluated the effects of CaCl_2_ on anthracnose disease caused by *ΔCsac1*. CaCl_2_ and both cAMP and CaCl_2_ failed to restore the ability to cause anthracnose disease in *ΔCsac1*, but restored that ability in the wild-type and *Csac1c* (**Figure 8A**). However, exogenous cAMP and CaCl_2_ restored penetration by *ΔCsac1* appressoria, as indicated by the presence of dendroid structures in the cuticle layer of pepper fruits (**Figure 8B**). This result suggests that CsAc1 is linked to the cAMP and Ca^2+^ signaling pathways in appressorium-mediated penetration by *C. scovillei*.

## Discussion

The cAMP signaling pathway is conserved and plays diverse roles in eukaryotic organisms (Lee and Dean, 1993; Shemarova, 2009; Zaccolo et al., 2021). During the early stage of fungal–plant interactions, pathogenic fungi recognize various physical and chemical signals from the host plant (Lee and Dean, 1993; Sadat et al., 2021). Intracellular cAMP transduces environmental signals to downstream functional targets to establish infection-related morphogenesis (Deising et al., 2000). Exogenous cAMP and the cAMP-dependent pathway are associated with host surface recognition and virulence in plant pathogenic fungi (Lee and Dean, 1993; Yang and Dickman, 1997; Mehrabi et al., 2009; Turrà et al., 2014; Zhu et al., 2017). Consistent with the foliar pathogens, our study showed that exogenous cAMP increased conidiation, appressorium formation, and pathogenicity of *C. scovillei* (**Figures 1B–E**). However, exogenous cAMP suppressed appressorium melanization of *C. scovillei* on hydrophilic and hydrophobic surfaces (**Figure 1B** and **Supplementary Table S2**), unlike *Colletotrichum higginsianum*, *C. lagenarium*, and *M. oryzae* (Lee and Dean, 1993; Yamauchi et al., 2004; Zhu et al., 2017). This result suggests a distinct effect of the cAMP signaling pathway in *C. scovillei*. We next characterized the role of the cAMP signaling pathway in *C. scovillei* using *ΔCsac1*, *ΔCscap1*, and *ΔCspdeh*. The intracellular cAMP concentration was confirmed to be significantly decreased in *ΔCsac1* and *ΔCscap1*, and increased in *ΔCspdeh*, compared to the wild-type (**Figure 2**).

**Figure 9:**
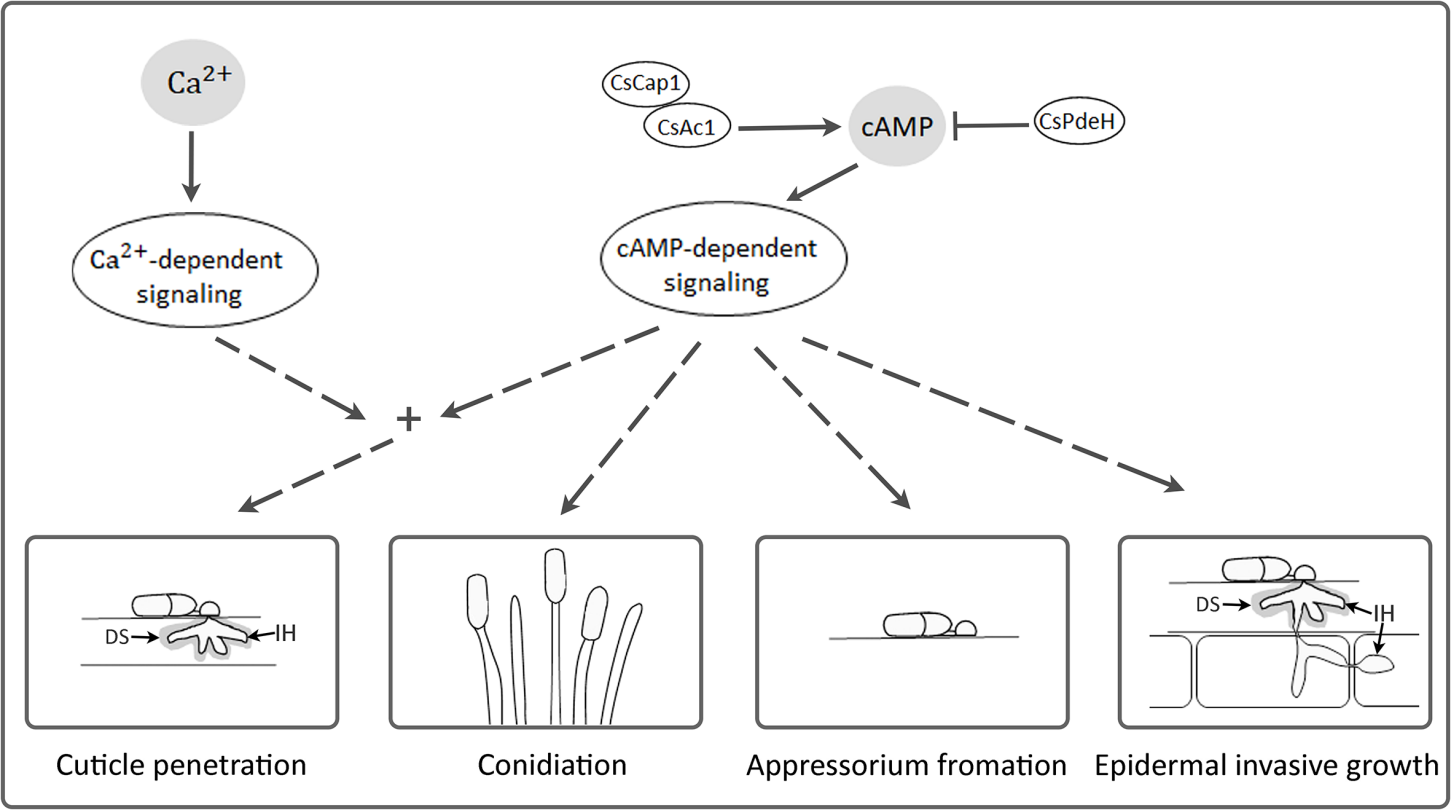
A proposed model of *CsAc1, CsCap1*, and *CsPdeH* in developments and pathogenicity of *C. scovillei*. Intracellular cAMP is positively regulated by CsAc1 and CsCap1, and negatively regulated by CsPdeH. The cAMP signaling pathway regulated conidiation, appressorium formation, cuticle penetration, and epidermal invasive growth of *C. scovillei*. The cAMP signaling pathway interconnects with Ca^2+^ signaling pathway and both regulate cuticle penetration of *C. scovillei*. DS and IH indicates dendroid structure and invasive hyphae, respectively.

The intracellular cAMP level is positively regulated by Ac1 with other regulators, including Cap1 (Purwin et al., 1986; Hurwitz et al., 1995; Zhou et al., 2012; Sun et al., 2022). Although *ΔCsac1* and *ΔCscap1* had similar intracellular cAMP levels and hyphal growth (**Figures 2**, **3**), our results indicate a distinct role of *CsAc1* and *CsCap1* in *C. scovillei*. *ΔCscap1* produced morphologically abnormal conidia, which are sensitive to thermal stress, whereas *ΔCsac1* formed morphologically normal conidia (**Figures 5A–D**). A role for *Cap1* in conidium morphology was also reported in *C. higginsianum* (Zhu et al., 2019). Given that the C-terminal domains of CsCap1 orthologs directly interact with actin monomers (Deeks et al., 2007; Zhou et al., 2012; Zhang et al., 2013), the morphologically abnormal conidia of *ΔCscap1* may be a result of a defect of actin reorganization, not a reduced intracellular cAMP level. Interestingly, deletion of *CsPdeH* resulted in failure of conidiation in *C. scovillei* (**Figures 4A–C**). In *Ustilaginoidea virens*, the *PdeH* deletion mutant dramatically reduced conidiation (Guo et al., 2019). The similar defect in conidiation was found in the double-deletion mutant *pdeHΔpdeLΔ* of *M. oryzae* (Ramanujam and Naqvi, 2010). It seems that intracellular cAMP over a particular threshold is detrimental to plant pathogenic fungi (Ramanujam and Naqvi, 2010). These results suggest that regulation of the intracellular cAMP level and cAMP-dependent signaling are associated with mycelial growth, conidiation, and conidial function in *C. scovillei* (**Figure 9**).

cAMP signaling regulates surface recognition and appressorium development (Lee and Dean, 1993; Choi and Dean, 1997; Yamauchi et al., 2004). Unexpectedly, *ΔCsac1* developed highly melanized appressoria that failed to penetrate the cuticle layers of pepper fruits (**Figure 7B**). However, the wild-type penetrated the host cuticle layer *via* non-melanized appressoria (**Figure 7B**). Melanin synthesis-related genes were upregulated in *ΔCsac1* compared to the wild-type (**Figure 7C**). This is consistent with the finding that exogenous cAMP suppressed appressorium melanization in the wild-type (**Figure 1B** and **Supplementary Table S2**). Although the mechanisms underlying the regulation by cAMP of appressorium melanization are unclear, it is possible that *C. scovillei* has evolved a different penetration strategy because non-melanized appressoria can penetrate the cuticle layer of pepper fruits (Fu et al., 2021). Moreover, our results indicate that *CsAc1* is important for the expression of cell wall-degrading enzymes, such as cutinases (**Figure 7D**). However, whether suppression of the melanin synthesis pathway is necessary to induce the expression of cutinases is unclear. Exogenous cAMP failed to restore the pathogenicity of *ΔCsac1* on pepper fruits (**Figure 8A**), suggesting *CsAc1* to be essential for the pathogenicity of *C. scovillei*. cAMP signaling is interconnected with Ca^2+^ signaling (Halls and Cooper, 2011; Chen et al., 2021). Ca^2+^ is implicated in appressorium development in filamentous fungi (Warwar and Dickman, 1996; Lee and Lee, 1998; Ahn et al., 2003; Uhm et al., 2003). For example, knockdown of Ca^2+^-permeable channel genes *via* RNA interference or deletion of *phospholipase C* genes (involved in the release of Ca^2+^ from cytosolic stores) resulted in defects in appressorium formation in *M. oryzae* (Nguyen et al., 2008; Rho et al., 2009; Choi et al., 2011). In this study, exogenous CaCl_2_ plus cAMP recovered appressorium-mediated penetration of *ΔCsac1*, as demonstrated by the presence of dendroid structures in the cuticle layer of pepper fruits (**Figure 8B**). This indicates that *CsAc1* and the Ca^2+^ signaling pathway are implicated in appressorium-mediated penetration in *C. scovillei* (**Figure 9**).

Downstream targets of cAMP signaling play diverse roles in stress responses, detoxification, ion homeostasis, secretion, and primary and secondary metabolism (Kronstad et al., 2011; Guo et al., 2016). In plant pathogenic fungi, downstream targets of the cAMP signaling pathway are associated with invasive growth (Adachi and Hamer, 1998; Mehrabi *et al*., 2009). In *M. oryzae*, deletion of *MAC1*, *Cap1*, or *PdeH* severely reduced invasive hyphal growth (Choi and Dean, 1997; Ramanujam and Naqvi, 2010; Zhou et al., 2012). However, *ΔCsac1* was completely defective in anthracnose formation on wounded pepper fruits (**Figure 7A**), suggesting that *CsAc1* is essential for invasive hyphal growth by *C. scovillei* (**Figure 9**). Unlike the failure of anthracnose development of *ΔCsac1*, *ΔCscap1* and *ΔCspdeh* caused anthracnose lesions of reduced severity on intact and wounded pepper fruits (**Figure 7A**). Indeed, invasive hyphal growth of *ΔCscap1* and *ΔCspdeh* in pepper epidermal cells was reduced compared to the wild-type (**Figure 7B**). These results suggest that the cAMP-dependent signaling pathway would be at least diverged into two aspects in pathogenicity and virulence in *C. scovillei*, which warrants further investigation.

Taken together, our findings show that cAMP positively regulates the development and pathogenicity of the pepper-fruit anthracnose pathogen *C. scovillei*. Conserved components of the cAMP signaling pathway, including *CsAc1, CsCap1*, and *CsPdeH*, are important for mycelial growth, conidiation, appressorium formation, and pathogenicity of *C. scovillei* (**Figure 9**). The cAMP signaling pathway is linked to the Ca^2+^ signaling pathway to regulate appressorium-mediated penetration of *C. scovillei* (**Figure 9**). These findings provide insight into the role of the cAMP signaling pathway in fruit anthracnose disease.

## Data availability statement

The original contributions presented in the study are included in the article/**Supplementary Material**. Further inquiries can be directed to the corresponding author.

## Author contributions

TF and KK conceived and designed the study. TF and H-HP performed experiment and analyzed data. TF and KK prepared the manuscript. All authors contributed to the article and approved the submitted version.

## Funding

This study was supported by the Basic Science Research Program through the National Research Foundation of Korea grant (NRF-2020R1A2C100550700) funded by the Ministry of Education, Science and Technology, and by a grant (918019043HD020) from the Strategic Initiative for Microbiomes in Agriculture and Food, Ministry of Agriculture, Food and Rural Affairs, Republic of Korea. The funders had no role in study design, data collection and analysis, decision to publish, or preparation of the manuscript.

## Conflict of interest

The authors declare that the research was conducted in the absence of any commercial or financial relationships that could be construed as a potential conflict of interest.

## Publisher’s note

All claims expressed in this article are solely those of the authors and do not necessarily represent those of their affiliated organizations, or those of the publisher, the editors and the reviewers. Any product that may be evaluated in this article, or claim that may be made by its manufacturer, is not guaranteed or endorsed by the publisher.
