# Supplementary material for: Role of the cAMP signaling pathway in the dissemination and development on pepper fruit anthracnose disease caused by Colletotrichum scovillei
Source: Front Cell Infect Microbiol. 2022 Oct 3;12:1003195. doi: 10.3389/fcimb.2022.1003195 (PMC9574036; doi:10.3389/fcimb.2022.1003195)
Supplement: Supplementary file 1 [file DataSheet_1.pdf]

Supplementary Material

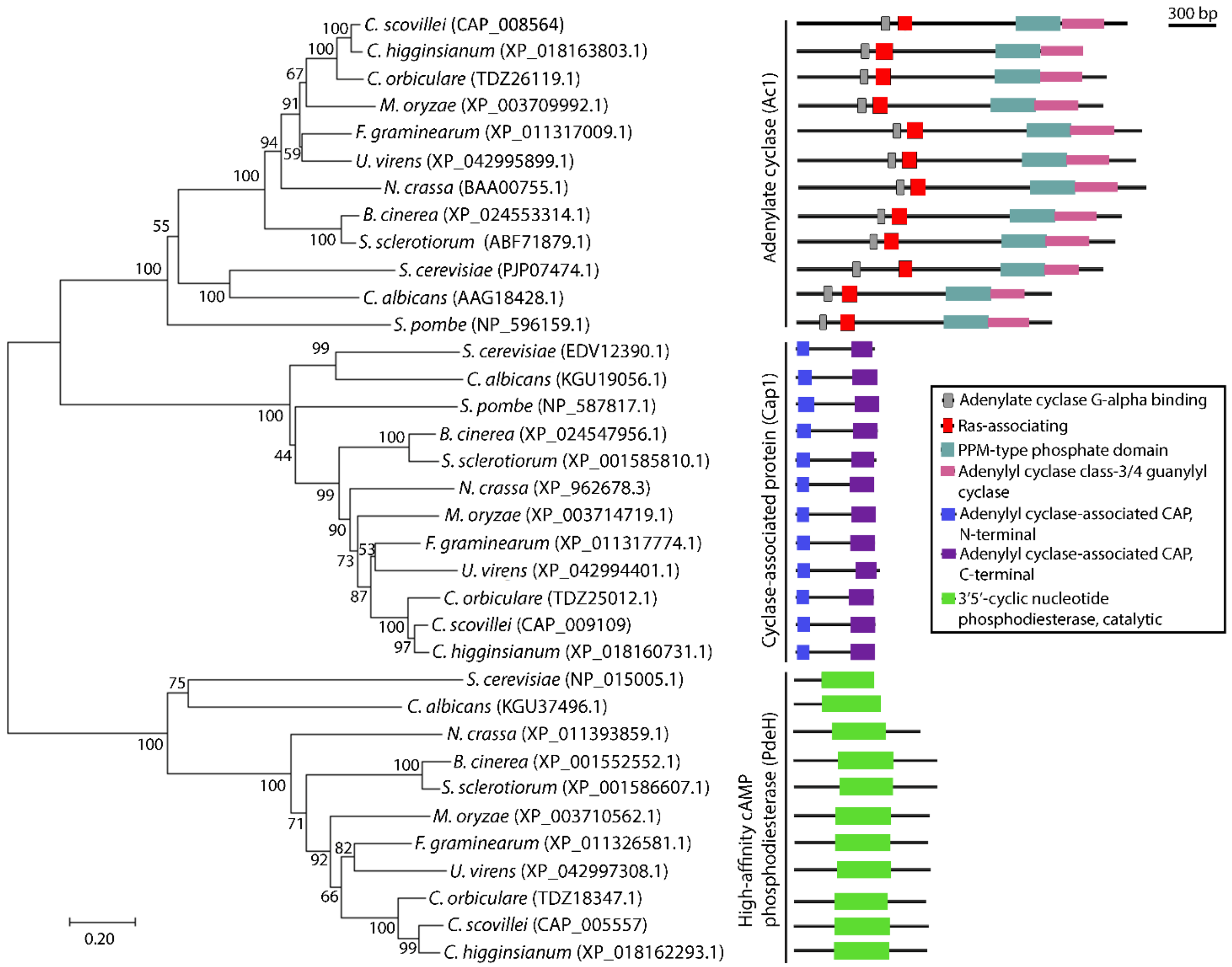

Supplementary Figure 1. Analysis of phylogenetic relationships and domain structures. Phylogenetic relationships were analyzed using the maximum-likelihood method with 1000 bootstraps in MEGA 7 software. Domain structures (IPR013716: adenylate cyclase G-alpha binding; IPR000159: Ras-associating domain; IPR001932: PPM-type phosphatase domain; IPR001054: adenylyl cyclase class-3/4/guanylyl cyclase; IPR013992: adenylate cyclase-associated CAP, N-terminal; IPR013912 adenylate cyclase-associated CAP, C-terminal; IPR002073: 3'5'-cyclic nucleotide phosphodiesterase, catalytic domain) were predicted using InterProScan.

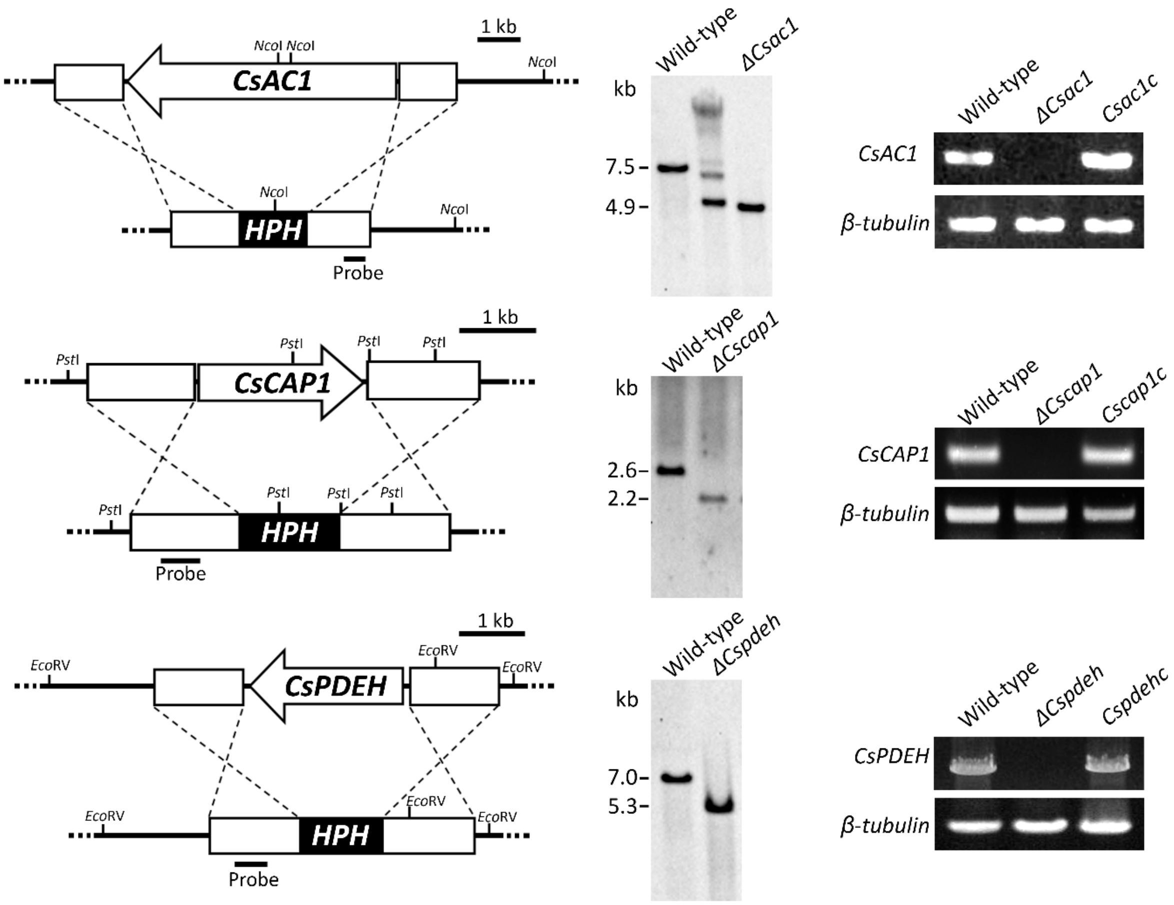

Supplementary Figure 2. Targeted deletion of *CsAc1*, *CsCap1*, and *CsPdeH*. An HPH cassette was used to replace each target gene (left panel). Each deletion mutant was confirmed using Southern blotting (middle panel). Expression of each target gene was detected in the wild-type and the corresponding complemented strain but not in the corresponding deletion mutant (right panel).

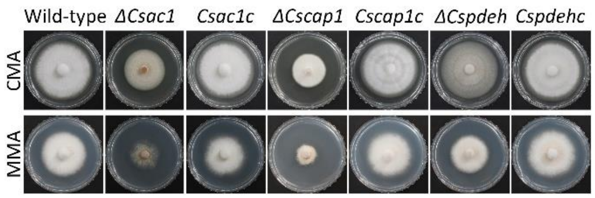

Supplementary Figure 3. Photographs of mycelial growth. Mycelial agar plugs (5 mm diameter) were inoculated onto CMA and MMA and incubated at 25°C in darkness for 5 days.

Supplementary Table 1. Primers used in this study.

Primers	Sequence (5'→3')
CsAc1 5F	CGCATCCGCAAACCTCATC
CsAc1 5R	CCTCCACTAGCTCCAGCCAAGCCCGAGACAGCATAACGTCTACCA
CsAc1 3F	GTTGGTGTCGATGTCAGCTCCGGAGTTCCCGAGAAACAAGGTTGAG
CsAc1 3R	GCCTTACCCAAAGGTCAAGTAA
CsAc1 NF	AGGACTCTTCCTCTTCCTCTT
CsAc1 NR	AGTAAGCACTACTTTGGGAATACA
CsAc1 SF	AACCAACCCTTCCGCATT
CsAc1 SR	CTGTTCCACACACCTCATCTAC
CsAc1 PF	GAATTGGAACAGCGACAGGA
CsAc1 PR	CATCCATAACGAACAGCAAAGC
CsAc1 RTF	CACAAAGCCCAGATTGATTCC
CsAc1 RTR	GAGAACCCAGAAGTCGTATACTC

CsCap1 5F	CGTGGTAGAAAGGGAGAAAG
CsCap1 5R	CCTCCACTAGCTCCAGCCAAGCCTCGACAGGAGAGGTTATAGG
CsCap1 3F	GTTGGTGTCGATGTCAGCTCCGGAGGAGTTGGGTCGATTTGTAGG
CsCap1 3R	TGCTACGTCGGGATTCA
CsCap1 NF	TAGTCGGTCCAAGTCCATT
CsCap1 NR	ATGTCATTAGCCAACACGAG
CsCap1 SF	TGAAACTTCACGCCCAATCT
CsCap1 SR	GGGTATGAAGATTACAGGGCAATA
CsCap1 PF	CGCTTCTTGGTTGCGTATCTA
CsCap1 PR	ATGTCATTAGCCAACACGAG
CsCap1 RTF	CGGAAATGACGCACAAGAAC
CsCap1 RTR	ATCAACGACAGACGAGTTGAG
CsPdeH 5F	GGAAACACTGTCCACCTGAT
CsPdeH 5R	CCTCCACTAGCTCCAGCCAAGCCAGATTTCCCAGCGATCGATATAC

CsPdeH 3F	GTTGGTGTCGATGTCAGCTCCGGAGCGTGATAAAGTCGTCGGGATT
CsPdeH 3R	ACTTTACGTCGCCACTTCAG
CsPdeH NF	CTTCGTCTGCGTCATGTCA
CsPdeH NR	GGTACAATGACCTCCCAACTC
CsPdeH SF	GTAGGCGAAGCACTCAAACA
CsPdeH SR	TATCAACAGCGCCAGCATAC
CsPdeH PF	GCGCAACATTTGTCTCACTC
CsPdeH PR	GCCGTGGTTGCTATGATTTG
CsPdeH RTF	CACAAAGCCCAGATTGATTCC
CsPdeH RTR	GAGAACCCAGAAGTCGTATACTC
**qRT-PCR**	
CsPKS1 qrtF	AGCTGCTTCTGGTGTTACTGCC
CsPKS1 qrtR	GCCCTTGTTGATCTCCTTCTTG
CsSCD1 qrtF	GCCGACAGCTACGACTCCAAGG

CsSCD1 qrtR	TCCCAGATCTTGTCGAGGAAGG
CsTHR1 qrtF	GTCGTCCAGGCCATCAAGAAGT
CsTHR1 qrtR	ACCAGAGTTGGAGCAGACAATG
CsCUT1 qrtF	AATCAGCGCTGGACCCAT
CsCUT1 qrtR	TCGTTGATGGCGACGGT
CsCUT2 qrtF	CCATCAACTATCCCGCATGT
CsCUT2 qrtR	CCCAGCATGACAATCTTCGT
CsCUT3 qrtF	CCCTGATTCAGTTAAGAGCAAGA
CsCUT3 qrtR	TGCCAGTGCACACCTTATC
CsCUT4 qrtF	GTTCCTCGTGCAGAAGCTC
CsCUT4 qrtR	TCGTTGAAGGAGGTTGAACTG
CsCUT5 qrtF	CACTCTTACTGAGCAAGCCATC
CsCUT5 qrtR	ATGACGGCACCTGCAAC
CsCUT6 qrtF	ATGGTCTTTGCTCGTGGTT

CsCUT6 qrtR	GCAAGAAGTTGGTGGAGAGA
CsCUT7 qrtF	TACTGCGACACGGGAGATA
CsCUT7 qrtR	GACGAACCGTTGGATGACTT
CsCUT8 qrtF	GGGCAACATTGCCATGAAT
CsCUT8 qrtR	GCTTGCTGGTTCAGAAGAGT
CsCUT9 qrtF	CGAGCTTGAGGCTTACTACA
CsCUT9 qrtR	CAGATCAAAGTGACCTTGCG
CsCUT10 qrtF	CCTGGCCAAATTCCCTTCT
CsCUT10 qrtR	GTGTCGATGCCGACAGAAT
CsCUT11 qrtF	CGCCTACATCAAGGAGAAGTG
CsCUT11 qrtR	GTGTAGTTCTCGGAAAGACCTG
CsCUT12 qrtF	ATCGTCCTCACTGGCTACA
CsCUT12 qrtR	AACTGAGCGGTAGCAACAC
CsCUT13 qrtF	TTGGCAAGGCAGACACTAC

CsCUT13 qrtR	CGTGGTTGATCCTCCTACAAC
CsCUT14 qrtF	CTGGAAGCGACAGCTACTTT
CsCUT14 qrtR	GCCAGCCAAGATATCTCCAA
CsCUT15 qrtF	CATCGCAATCCAGGGAGTAAA
CsCUT15 qrtR	TAGCCCGAGACCGTGAG
CsCUT16 qrtF	GATGCCCAGAAGAACCAGAT
CsCUT16 qrtR	TTTCCGTCGCAGACAGTG
CsCUT17 qrtF	GGTTCTAGAGACGTGGTCAAC
CsCUT17 qrtR	CGATTTGATGCGGCCATAGA
CsCUT18 qrtF	GGCACGGGAGTCATTGG
CsCUT18 qrtR	CTGGACCAGCTTCGTCATC

Supplementary Table 2. Appressorium melanization of the wild-type (strain KC05) with exogenous cAMP.

cAMP (µM)	Melanized appressoria (%)	
	Hydrophobic surface	Hydrophilic surface

0	96.9±1.7a	97.6±1.4a
5	90.6±3.0b	85.0±6.7b
50	76.3±5.5c	69.1±4.9c
500	25.0±7.7d	20.3±3.7d
2500	4.3±3.3e	3.2±2.9e

A conidial suspension (5 × 10^4/mL) of the wild-type was dropped onto the hydrophobic coverslips and hydrophilic slide glasses and incubated in a humid plastic box. After 12 hours, exogenous cAMP was added to the conidial drops. Melanized appressoria were counted among 100 appressoria at 24 hours after inoculation. Different letters in the same group indicate significant differences estimated using Duncan's test ($P < 0.05$).

Supplementary Table 3. Predicted cutinases in the genome of *C. scovillei* KC05 strain.

**Locus**	**Designated name**	**Length (aa)**	**Domain**
CAP_007018	CsCUT1	227	IPR000675
CAP_001807	CsCUT2	333	IPR000675
CAP_002100	CsCUT3	238	IPR000675
CAP_002133	CsCUT4	227	IPR000675
CAP_003059	CsCUT5	352	IPR000675
CAP_004758	CsCUT6	219	IPR000675

CAP_004776	CsCUT7	238	IPR000675
CAP_004905	CsCUT8	277	IPR000675
CAP_004925	CsCUT9	227	IPR000675
CAP_006261	CsCUT10	249	IPR000675
CAP_007007	CsCUT11	286	IPR000675
CAP_007500	CsCUT12	204	IPR000675
CAP_009465	CsCUT13	333	IPR000675
CAP_010115	CsCUT14	240	IPR000675
CAP_010717	CsCUT15	327	IPR000675
CAP_011102	CsCUT16	226	IPR000675
CAP_011103	CsCUT17	228	IPR000675
CAP_011452	CsCUT18	233	IPR000675
